# Optimizing intraocular lens power calculation using adjusted conventional keratometry for cataract surgery combined with Descemet membrane endothelial keratoplasty

**DOI:** 10.1007/s00417-022-05598-6

**Published:** 2022-03-08

**Authors:** Raphael Diener, Maximilian Treder, Jost Lennart Lauermann, Nicole Eter, Maged Alnawaiseh

**Affiliations:** 1 Department of Ophthalmology, University of Muenster Medical Center, Albert-Schweitzer-Campus 1, Building D15, 48149 Muenster, Germany; 2 Department of Ophthalmology, Fulda Medical Center, Fulda, Germany

**Keywords:** Hyperopic shift, Conventional keratometry, DMEK, Triple DMEK, IOL power calculation, PA ratio, Posterior to anterior corneal curvature radii ratio, Adjusted keratometry, Conventional keratometry

## Abstract

**Purpose:**

To evaluate the utility of intraocular lens (IOL) power calculation using adjusted conventional keratometry (K) according to postoperative posterior to preoperative anterior corneal curvature radii (PPPA) ratio for eyes with Fuch’s dystrophy undergoing cataract surgery combined with Descemet membrane endothelial keratoplasty (triple DMEK).

**Methods:**

A fictitious refractive index (FRI) was determined (Pentacam HR®) based on the PPPA ratio in 50 eyes undergoing triple DMEK. Adjusted corneal power was calculated in every eye using adjusted K values: K values determined by the IOLMaster were converted to adjusted anterior corneal radius using the mean FRI. Posterior corneal radius was calculated using the mean PPPA ratio. Adjusted corneal power was determined based on the calculated corneal radii and thick lens formula. Refractive errors calculated using the Haigis, SRK/T, and HofferQ formulae based on the adjusted corneal power were compared with those based on conventional K measurements.

**Results:**

Calculated PPPA ratio and FRI were 0.801 and 1.3271. Mean prediction error based on conventional K was in the hyperopic direction (Haigis: 0.84D; SRK/T: 0.74D; HofferQ: 0.74D) and significantly higher (*P* < 0.001) than that based on adjusted corneal power (0.18D, 0.22D, and 15D, respectively).

When calculated according to adjusted corneal power, the percentage of eyes with a hyperopic shift > 0.5D fell significantly from 64 to 30% (Haigis), 62 to 36% (SRK/T), and 58 to 26% (HofferQ), respectively.

**Conclusion:**

IOL power calculation based on adjusted corneal power can be used to reduce the risk of a hyperopic shift after triple DMEK and provides a more accurate refractive outcome than IOL power calculation using conventional K.
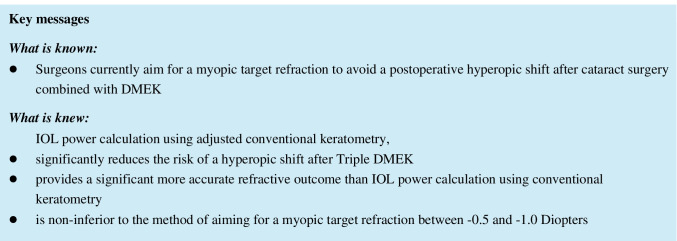

**Supplementary Information:**

The online version contains supplementary material available at 10.1007/s00417-022-05598-6.

## Introduction

Descemet membrane endothelial keratoplasty (DMEK) is frequently used for the surgical treatment of endothelial diseases, such as Fuch’s endothelial corneal dystrophy (FECD) [1]. Posterior lamellar keratoplasty has prevailed over penetrating keratoplasty due to faster visual rehabilitation, a lower risk of graft failure and improved safety profile [2, 3]. DMEK can also be safely performed simultaneously with phacoemulsification and intraocular lens (IOL) implantation in patients with clinically significant cataract (triple DMEK) [4].

There are two main problems concerning the postoperative refractive outcome after triple *DMEK.*

First, several studies report an average postoperative hyperopic shift of approximately + 0.5 to + 1.0 diopters (D) after triple DMEK [4–8]. To counteract this, surgeons aim for a more myopic target refraction of − 0.5D to − 1.0D [4–6]. Second, despite this adjustment, patient-individual variations in the postoperative refractive outcome ranging from hyperopia to myopia are observed [6, 8]. Attempts have therefore been made to estimate the degree of the hyperopic shift after triple DMEK in every patient on the basis of preoperative corneal parameters and to take these into account when selecting the target refraction [6, 9]. However, no attempt has been made so far to optimize the erroneous IOL power calculation in eyes with Fuch’s dystrophy undergoing triple DMEK.

The four potential errors of IOL power calculation lie in corneal curvature measurement, axial length (AL) measurement, effective lens position (ELP) estimation, and the calculation formula used [10–12]. In the eyes undergoing triple DMEK, IOL power calculation using conventional keratometry (K) to measure corneal power is invalid [13], due to a steeper posterior corneal curvature in eyes after DMEK compared to healthy eyes [13–15]. The aim of this study was to evaluate and optimize IOL power calculation using adjusted keratometry values based on the postoperative posterior to preoperative anterior corneal curvature radii (PPPA) ratio [13] for the eyes with Fuch’s endothelial corneal dystrophy undergoing triple DMEK.

## Patients and methods

This retrospective study was approved by the local institutional review board (Ethics Committee of the WWU Muenster, Germany) and adhered to the tenets of the Declaration of Helsinki.

Fifty eyes of fourty-two patients with Fuch’s dystrophy who underwent uncomplicated phacoemulsification combined with Descemet membrane endothelial keratoplasty in the Department of Ophthalmology of the University Hospital of Muenster were included in this study.

Eyes with a history of other corneal diseases, corneal infection or intraocular inflammation, trauma, corneal scars, contact lens worn 4 weeks before measurement, clinically significant graft detachment, or delayed corneal clearance were excluded.

### Surgical procedure

On the preoperative day, a Nd:YAG iridotomy was performed at six o’clock positions. We obtained corneas from the cornea bank as corneoscleral disks and grafts were stored in commercially available organ culture media (Biochrom, Berlin, Germany). Grafts were prepared using the technique of *stripping from the trabecular meshwork* [16, 17]. After uneventful cataract surgery the 8.75–9.00-mm donor Descemet roll was stained with a 0.06% trypan blue solution (Vision Blue, D.O.R.C. International) and sucked in to a glass injector (DMEK-Inserter, Geuder, Germany) for injection into the anterior chamber. To position the graft onto the recipient posterior stroma, air or gas (SF6 20%) was injected underneath the graft. After surgery, patients were asked to maintain a supine position for at least 4 h.

### IOL power calculation with conventional keratometry

A partial coherence interferometry (PCI) device (IOL Master 500; version 7.3; Carl Zeiss Meditec, Jena, Germany) was used for measurement of AL, ACD, and anterior corneal radii. Conversion of corneal radii to power was performed with a keratometric index of 1.3320. Surgeons aimed for a target refraction ranging between − 0.5 and − 1.0 D (Haigis: − 0.69 D ± 0.38; SRK/T: − 0.59 D ± 0.42; HofferQ: − 0.59 D ± 0.36). Postoperative refraction was measured once after refractive stability had returned (a minimum of 3 months after surgery) [18].

### IOL power calculation with adjusted keratometry

Calculation of the IOL power and predicted refraction was performed with adjusted keratometry values using the Haigis, SRK/T, and HofferQ formulae. Conventional keratometry was adjusted using the PPPA ratio and FRI based on pre- and postoperative measurements.

A detailed way to calculate adjusted keratometry based on the PPPA ratio and FRI can be found in Supplementary files 1 and 2.

### Refractive prediction error

The median absolute error (MedAE) was defined as the median absolute value of the refractive prediction error. The mean absolute error (MAE) was defined as the mean absolute value of the refractive prediction error. The refractive prediction error (PE) was defined as the difference between the postoperative refractive spherical equivalent and the preoperative predicted refraction determined using the Haigis, SRK/T, and HofferQ formulae with conventional keratometry and adjusted keratometry dependent on the power of the implanted IOL.

Furthermore, we calculated the MedAE, MAE, and PE after subtracting the hyperopic shift which was anticipated by the surgeon as seen in Table 2, which is referred to as *conventional keratometry modified*. To calculate this, the target refraction chosen by the surgeon is subtracted from the postoperative spherical equivalent.

### Statistics

Microsoft Excel 2010 was used for data management. Statistical analyses were performed with IBM SPSS® Statistics 22 for Windows (IBM Corporation, Somers, NY, USA). The normality of the data distribution was tested using the Kolmogorov–Smirnov test. Depending on the normality distribution, data were compared using the paired *t*-test or two-sided Wilcoxon signed-rank test. An exact chi-quadrat test was used to test differences between categorical variables. The data are presented as mean ± standard deviation (SD). The median absolute error (MedAE) values are presented as median [25, 75 percentiles]. Interferential statistics are intended to be exploratory (hypotheses-generating), not confirmatory, and are interpreted accordingly. The comparison-wise type-I error rate is controlled instead of the experiment-wise error rate. The local significance level was set at *P* ≤ 0.05.

## Results

Postoperative measurements were performed 12 months after surgery in average. The demographic data of the study population are shown in Table 1. The mean prediction error (PE) based on conventional keratometry (42.86 ± 1.72 D) was in the hyperopic direction (Haigis: 0.84 D, SRK/T: 0.74 D, HofferQ: 0.74 D).

**Table 1:** Demographic, biometric, and tomographic data of the study population

Parameter	Mean ± SD
Age, (Y)	67 ± 8
Sex, (M:F)	26:24
Laterality, (R:L)	26:24
IOL, (Zeiss Asphina:CT Lucia)	43:7
IOLMaster 500®	
IOL power, (D)	22.60 ± 2.46
ACD, (mm)	3.09 ± 0.34
AL, (mm)	23.63 ± 0.92
K1, (D)	42.06 ± 1.96
K2, (D)	43.55 ± 1.63
Conventional Keratometry, (D)	42.86 ± 1.72
Pentacam HR®	
PPPA ratio	0.801 ± 0.04
FRI	1.3271 ± 0.0001
Adjusted keratometry (D)^1^	42.91 ± 1.72

Legend: *SD* standard deviation, *Y* years, *M* male, *F* female, *R* right, *L* left, *D* dioptres, *IOL power* power of the implanted intraocular lens, *Zeiss Asphina* Zeiss Asphina 409 M, *CT Lucia* CT Lucia 211P (both: Carl Zeiss Meditec, Jena, Germany), *ACD* anterior chamber depth, *mm* millimeter, *AL* axial length, *K1* horizontal keratometric readings, *K2* vertical keratometric readings; *PPPA ratio* postoperative posterior to preoperative anterior corneal curvature radii ratio, *FRI* fictitious refractive index, ^1^Mean adjusted keratometry calculated using the PPPA and FRI index

### Adjusted keratometry values

The Pentacam HR® values required for the calculation of adjusted conventional keratometry were as follows: anterior corneal radius (R_A_) flattened (preoperative: 7.81 ± 0.32 mm, postoperative: 7.88 ± 0.32 mm; *P* = 0.017), whereas posterior corneal radius (R_B_) steepened significantly from 7.06 ± 0.71 to 6.26 ± 0.35 mm (*P* < 0.01), leading to a mean PPPA ratio of 0.801 ± 0.04. With the thick lens formula, the calculated mean FRI of the group of patients studied was 1.3271 ± 0.0001. Using the PPPA ratio and FRI the mean adjusted keratometry was 42.91 ± 1.72 D with an average difference of 0.05 ± 0.01 D between adjusted corneal power and conventional keratometer values.

### Refractive outcomes

Using adjusted keratometry the PE was significantly (all *P* < 0.001) lower than that based on conventional K (Haigis: 0.18 D; SRK/T: 0.22 D; HofferQ: 0.15 D). The median arithmetic error (MedAE) based on conventional keratometry and calculated using the Haigis, SRK/T, and HofferQ formulae (Haigis: 0.78 D; SRK/T: 0.70 D, HofferQ: 0.63 D) was higher than that based on adjusted corneal power (Haigis: 0.67 D, *P* = 0.003; SRK/T: 0.60, *P* = 0.02; HofferQ: 0.49 D, *P* = 0.004).

The percentage of the eyes with a hyperopic shift > 0.5 D calculated with adjusted corneal power was reduced significantly, indeed almost halved from 64%, 62%, and 58% to 30% (*P* < 0.001), 36% (*P* < 0.01), and 26% (*P* < 0.001) with Haigis, SRK/T, and HofferQ, respectively. The refractive outcomes obtained using the Haigis, SRK/T, and HofferQ formulae are summarized in Table 2.

**Table 2:** Comparison of PE, MedAE, and MAE based on the PPPA ratio between calculations using conventional K and those using adjusted corneal power in the Haigis, SRK/T, and HofferQ formulae

	Formula	Conventional K (1)	Conventional KM (2)	*P* (1 vs. 2)	Adjusted K (3)	*P* (1 vs. 3)
PE, D^*^	Haigis	0.84 ± 0.97	0.15 ± 0.98	< 0.001^2^	0.18 ± 0.96	< 0.001^2^
	SRK/T	0.74 ± 0.92		< 0.001^2^	0.22 ± 0.98	< 0.001^2^
	HofferQ	0.74 ± 0.93		< 0.001^2^	0.15 ± 0.91	< 0.001^2^
MedAE, D^†^	Haigis	0.78 [0.49, 1.55]	0.63 [0.28; 0.96]	0.003^1^	0.67 [0.20, 1.21]	0.003^1^
	SRK/T	0.70 [0.47, 1.19]		0.020^1^	0.60 [0.27, 1.06]	0.020^1^
	HofferQ	0.63 [0.45, 1.19]		0.05^1^	0.49 [0.16, 1.04]	0.004^1^
MAE, D^*^	Haigis	1.02 ± 0.79	0.76 ± 0.64	0.009^2^	0.76 ± 0.61	0.004^2^
	SRK/T	0.93 ± 0.73		0.05^2^	0.77 ± 0.65	0.04^2^
	HofferQ	0.92 ± 0.74		0.05^2^	0.69 ± 0.61	0.002^2^
± PE, *n* (%)						
± 0.25 D, *n* (%)	Haigis	9 (18%)	8 (16%)		14 (28%)	
	SRK/T	7 (14%)			11 (22%)	
	HofferQ	8 (16%)			15 (30%)	
± 0.5 D, *n* (%)	Haigis	13 (26%)	15 (30%)		20 (40%)	
	SRK/T	16 (32%)			20 (40%)	
	HofferQ	16 (32%)			26 (52%)	
± 1.0 D, *n* (%)	Haigis	31 (62%)	37 (74%)		32 (64%)	
	SRK/T	32 (64%)			35 (70%)	
	HofferQ	31 (62%)			34 (68%)	
± 1.5 D, *n* (%)	Haigis	36 (72%)	45 (90%)		43 (86%)	
	SRK/T	40 (80%)			42 (84%)	
	HofferQ	38 (76%)			44 (88%)	
± 2.0 D, *n* (%)	Haigis	42 (84%)	47 (94%)		48 (96%)	
	SRK/T	44 (88%)			46 (92%)	
	HofferQ	44 (88%)			48 (96%)	
> PE, *n* (%)						
> + 0.5D, *n* (%)	Haigis	32 (64%)	17 (34%)	0.002^3^	15 (30%)	0.0006^3^
	SRK/T	31 (62%)		0.005^3^	18 (36%)	0.009^3^
	HofferQ	29 (58%)		0.01^3^	13 (26%)	0.001^3^

Legend: *Conventional K* conventional keratometry, *Conventional KM* conventional keratometry modified, *Adjusted K* adjusted keratometry, *P P*-value, *n* number, *PE* prediction error, *MedAE* median absolute error, *MAE* mean absolute error, *D* dioptres, ^1^Wilcoxon signed-rank test, ^2^paired *t*-test, ^3^X-square test, *significant P-values* bolt, †data are presented as median [interquartile range], *data are presented as mean (standard deviation), % percent

## Discussion

A standard IOL power calculation for eyes undergoing cataract surgery combined with DMEK is prone to error. Mean refractive outcomes deviate significantly in the hyperopic direction between + 0.50 and + 1.00 from the planned refraction depending on the study [4–8]. Similarly, in our data, a hyperopic shift with an average of + 0.7 D was present.

IOL power is traditionally calculated from on keratometer readings that estimate the corneal refractive power from anterior corneal measurements alone [19]. This so-called conventional K is used in IOL Master PCI devices and assumes a normal and constant PA ratio in all eyes [19].

In our study, the posterior corneal curvature was found to change significantly after DMEK, a finding in line with results presented in the literature [18, 20, 21]. Therefore, as the posterior corneal curvature is not measured directly but changes significantly after DMEK, the decisive ratio for IOL power calculation prior to triple DMEK is the postoperative posterior (once stable refraction has been achieved) to the preoperative anterior (when conventional K is performed) corneal curvature radii (PPPA) ratio.

However, the PPPA ratio in Fuch’s dystrophy eyes undergoing triple DMEK is significantly different to the PA ratio in healthy eyes and therefore renders conventional K and the corneal power derived from it by this method invalid, as was confirmed in a previous study by our group [13].

Similar results are reported in other studies. Arnalich-Montiel et al. found a thinner central corneal thickness, with a steeper pachymetric progression to the periphery in the eyes after DMEK compared to normal eyes using Scheimpflug imaging [15]. Similarly, Hayashi et al. found a steeper posterior corneal curvature in comparison to healthy controls using optical coherence tomography [14].

Thus, using conventional K for corneal power estimation in eyes undergoing triple DMEK leads to an underestimation of the postoperative (more negative) posterior corneal power and hence an overestimation of the total corneal power and the resulting IOL power is underestimated, so that patients are likely to experience postoperative hyperopia [13].

There are numerous methodologies that aim to overcome the problem of altered PA ratios by modifying the keratometric index [22–24]. Kim et al. introduced *Eom’s adjustment method* [25] in 2018. This method suggests an adjustment of IOL Master K values according to the patient’s individual preoperative PA ratio without changing the mean value of the entire data set. In their method, adjustment of K values is based on the preoperative PA ratio and a fictitious refractive index calculated with a thick lens formula, determined using Scheimpflug imaging (Pentacam HR®) [25].

Similarly, the present study optimizes the IOL calculation in eyes undergoing triple DMEK using adjusted K values.

The proposed method significantly reduced the percentage of patients developing a postoperative hyperopic shift. Furthermore, we significantly improved the refractive prediction accuracy of IOL power calculation by using the Haigis, SRK/T, and HofferQ formulae in eyes undergoing triple DMEK. Also, there is no significant difference of the introduced methodology to the current gold standard of simply choosing the target refraction in between the range of − 0.5 and − 1.0 D as seen in Table 2.

**Fig. 1:**
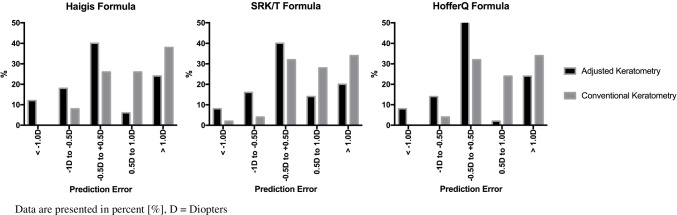
Refractive error distribution using conventional K and adjusted conventional K values for IOL power calculation in the Haigis, SRK/T, and HofferQ formula

However, the postoperative refractive outcome still ranges from hyperopia to myopia, as seen in Fig. 1, leading to a high standard deviation of the prediction error (± 0.90).

**Fig. 2:**
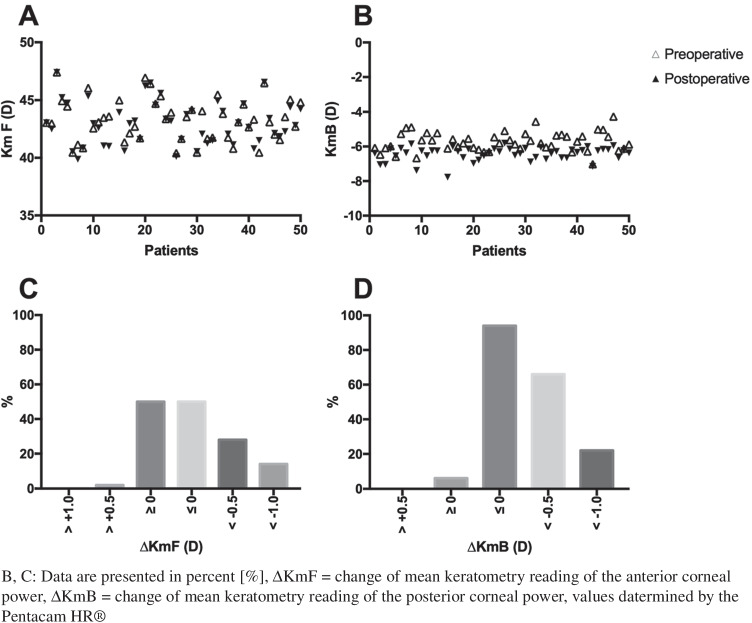
Pre- and postoperative anterior (**A**) and posterior corneal power (**B**) and the distribution of the resulting dioptric change (**C**, **D**)

In the present study, this might be explained by a change of the anterior corneal curvature as seen in Fig. 2. Using adjusted keratometry values addresses the problem of the steeper postoperative corneal curvature; however, the problem of patient-individual refractive outcomes due to possible changes of the anterior corneal radius or other causes remains unsolved.

As both corneal and surgical parameters such as descemetorhexis size, Descemet roll size, and both steepness and location of the incision could affect the PPPA ratio, an individualized PPPA ratio may need to be calculated and optimized over time for different surgeons and patients to improve refractive outcome. To calculate a FRI for a specific patient group at risk of a myopic or high hyperopic shift, the larger number of patients is required.

## Conclusion

In conclusion, IOL power calculation using adjusted corneal power based on the PPPA ratio might predict postoperative refraction more accurately than that obtained through conventional K in eyes undergoing triple DMEK and reduces the risk of a postoperative hyperopic shift in these eyes. The new methodology is non-inferior to the current gold standard of choosing a target refraction between − 0.5 and − 1.0 D.

## Supplementary Information

Below is the link to the electronic supplementary material.

Supplementary file1 (TIFF 1318 KB)

Supplementary file2 (DOCX 16 KB)

Supplementary file3 (DOCX 12 KB)
