# Supplementary figures and images for: Optimizing intraocular lens power calculation using adjusted conventional keratometry for cataract surgery combined with Descemet membrane endothelial keratoplasty
Source: Graefes Arch Clin Exp Ophthalmol. 2022 Mar 8;260(9):3087–93. doi: 10.1007/s00417-022-05598-6 (PMC9418294; doi:10.1007/s00417-022-05598-6)

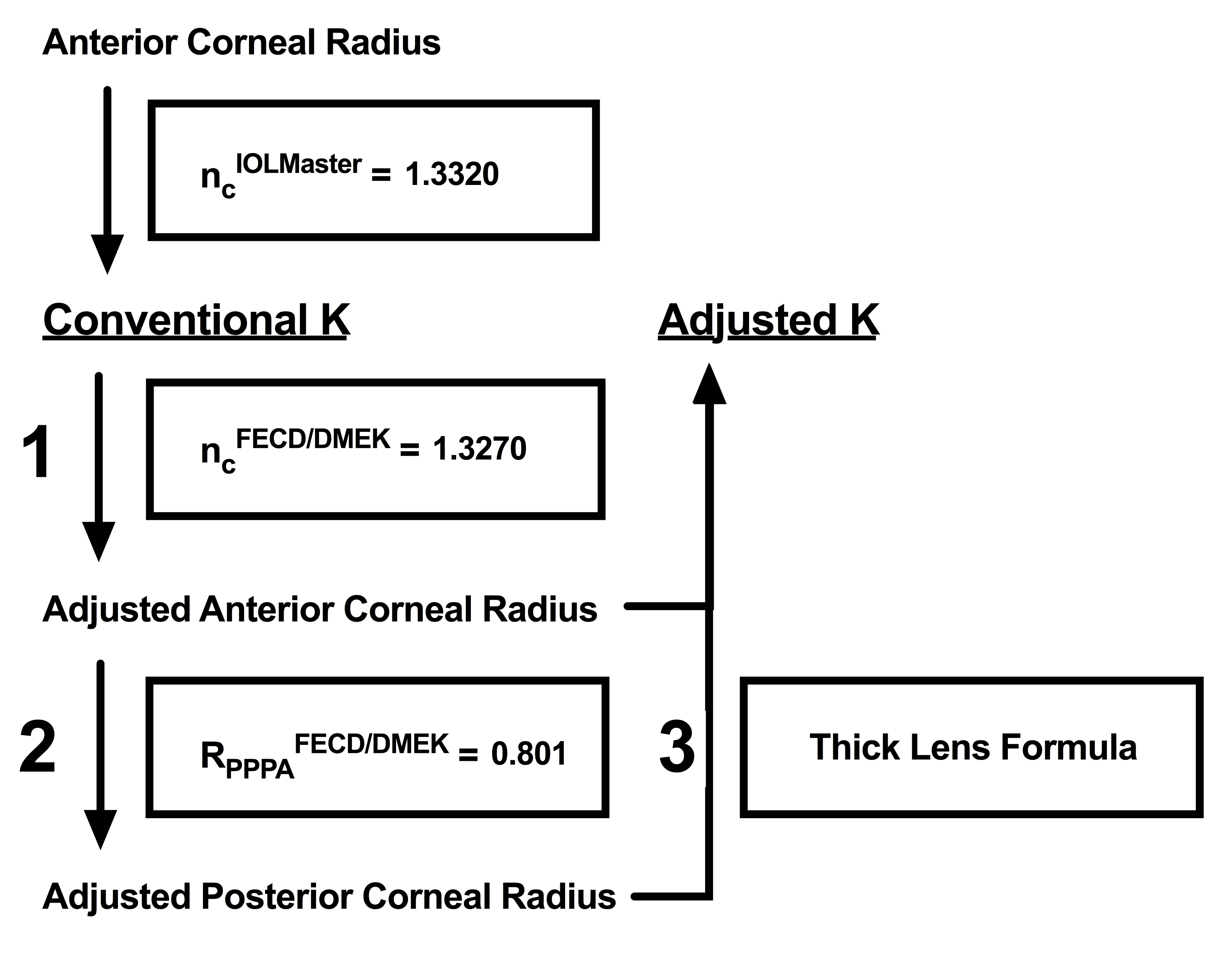

Supplement: Supplementary file 1 — Supplementary file1 (TIFF 1318 KB) [file 417_2022_5598_MOESM1_ESM.tiff]
