# Supplementary material for: Optimizing intraocular lens power calculation using adjusted conventional keratometry for cataract surgery combined with Descemet membrane endothelial keratoplasty
Source: Graefes Arch Clin Exp Ophthalmol. 2022 Mar 8;260(9):3087–93. doi: 10.1007/s00417-022-05598-6 (PMC9418294; doi:10.1007/s00417-022-05598-6)
Supplement: Supplementary file 2 — Supplementary file2 (DOCX 16 KB) [file 417_2022_5598_MOESM2_ESM.docx]

**Supplementary File 1**

**Calculation of the PPPA ratio**

First, the ratio between preoperative anterior corneal radius and postoperative posterior corneal radius (PPPA ratio or R_PPPA_^FECD/DMEK^) was calculated in every eye based on the preoperative anterior corneal radius (R_A_^FECD^) and postoperative posterior corneal radius (R_P_^DMEK^). (Formula 1)

| $R_{{PPPA}^{FECD/DMEK}}= \frac{R_{A^{FECD}}}{R_{P^{DMEK}}}$ | ( 1 ) |
| --- | --- |

The mean PPPA ratio was then calculated based on all eyes included in the study.

**Calculation of the FRI for a single Scheimpflug camera.**

Second, a fictitious refractive index (FRI) was calculated for every eye based on the thick lens formula and the postoperative posterior to preoperative anterior corneal curvature radii ratio.

|  | $D_{A^{FECD}}=\frac{{(n}_{Cornea}-1) x 1000}{R_{A^{FECD}}}$ | (2) |
| --- | --- | --- |
|  | $D_{P^{DMEK}}=\frac{{(n}_{aqueous}-n_{cornea}) x 1000}{R_{A^{FECD}} x R_{{PPPA}^{FECD/DMEK}}}$ | (3) |
|  | $D_{T^{FECD/DMEK}}=\frac{{Mean CCT}^{DMEK}}{n_{cornea}x 1000} x D_{A^{FECD}}x D_{P^{DMEK}}$ | (4) |
|  | $D_{{Total}^{FECD/DMEK}}=D_{A^{FECD}}+ D_{P^{DMEK}}- D_{T^{DMEK}}$ | (5) |
|  | $n_{c}=\frac{D_{{Total}^{FECD/DMEK}} {x R}_{A^{FECD}}}{1000}+1$ | (6) |

*D_A_*  is the dioptric power of the anterior corneal surface (D_A_^FECD^ = preoperative; D_A_^DMEK^ = postoperative), *n_cornea_* is the refractive index of the cornea (1.376), *D_p_* is the dioptric power of the posterior corneal surface (D_P_^FECD^ = preoperative; D_P_^DMEK^ = postoperative), R_A_ is the mean anterior corneal radius (R_A_^FECD^ = preoperative; R_A_^DMEK^ = postoperative) *n_aqueous_* is the refractive index of aqueous (1.336), mean *R_PPPA_^FECD/DMEK^* is the mean postoperative posterior to preoperative anterior corneal curvature radii ratio of all eyes and *D*_Total_^FECD/DMEK^ is the dioptric power of the cornea, while D_T_^FECD/DMEK^ is the mean dioptric power of the central corneal thickness, mean CCT^DMEK^ is the mean postoperative central corneal thickness. Finally, the mean FRI was calculated based on all eyes included in this study.

**Adjustment of Conventional K**

Anterior corneal radii were measured using the IOLMaster 500 and were converted to dioptric power of conventional K measurements with a keratometric index of 1.3320. The conversion of conventional K to adjusted conventional K values followed a rule of three (see **figure 2**): First (1.): The conventional K values were calculated back to adjusted anterior corneal radius (r_A_), using the mean FRI (n_c_) (Formula 7). Second (2.): Adjusted anterior corneal radius (r_A_) was converted to adjusted posterior corneal radius (r_P_) using the mean PPPA ratio (Formula 8). Third (3.): Adjusted corneal power was calculated using a thick lens formula based on the adjusted anterior corneal curvature radius (r_A_), predicted posterior corneal curvature radius (r_P_), refractive indices of cornea and aqueous humor (1.376 and 1.336, respectively), and mean postoperative central corneal thickness.

| $r_{A}=\frac{{(n}_{c^{FECD/DMEK}}-1) x 1000}{D_{IOL Master}}$ | (7) |
| --- | --- |
| $r_{P}=r_{A}x R_{PPPA}$ | (8) |

r_A_ is the adjusted anterior corneal curvature radius, n_c_^FECD/DMEK^ is the mean FRI, D_IOLMaster_ is the dioptric power of K measurements of the IOLMaster, r_P_ is the predicted posterior corneal curvature radius, and R_PPPA_ is the mean PPPA ratio
