# Supplementary material for: Optimizing intraocular lens power calculation using adjusted conventional keratometry for cataract surgery combined with Descemet membrane endothelial keratoplasty
Source: Graefes Arch Clin Exp Ophthalmol. 2022 Mar 8;260(9):3087–93. doi: 10.1007/s00417-022-05598-6 (PMC9418294; doi:10.1007/s00417-022-05598-6)
Supplement: Supplementary file 3 — Supplementary file3 (DOCX 12 KB) [file 417_2022_5598_MOESM3_ESM.docx]

**Supplementary file 2**

**Figure 1 (supplementary file): Calculation of adjusted corneal power using a rule of three**

Legend to figure 1: The conversion of conventional K to adjusted conventional K values follows a rule of three: First (1): The calculated conventional K values are calculated back to adjusted anterior corneal radius (r_A_), using the fictitious keratometer index (n_c_). Second (2): Adjusted anterior corneal radius (r_A_) is converted to adjusted posterior corneal radius (r_P_) using the PPPA ratio. Third (3.): Adjusted corneal power is calculated using a thick lens formula based on the adjusted anterior corneal curvature radius (r_A_), predicted posterior corneal curvature radius (r_P_), refractive indices of cornea and aqueous humor (1.376 and 1.336, respectively), and mean postoperative central corneal thickness.
